# A Study of Rainfall-Runoff Movement Process on High and Steep Slopes Affected by Double Turbulence Sources

**DOI:** 10.1038/s41598-020-66060-3

**Published:** 2020-06-02

**Authors:** Xinghua Li, Jianen Gao, Zihao Guo, Yan Yin, Xingchen Zhang, Pengcheng Sun, Zhe Gao

**Affiliations:** 1 College of Water Resources and Architectural Engineering, Northwest A&F University, Yangling, Shaanxi 712100 China; 2 Institute of Soil and Water Conservation, Northwest A&F University, Yangling, Shaanxi 712100 China; 3 Institute of Soil and Water Conservation, Chinese Academy of Sciences and Ministry of Water Resources, Yangling, Shaanxi 712100 China; 4 Research Center on Soil & Water Conservation Ministry of Water Resources, Yangling, 712100 China

**Keywords:** Environmental impact, Hydrology

## Abstract

To increase the available land area, a large-scale land remediation campaign was carried out in the loess hilly and gully area. A large number of high and steep slopes have been produced in the construction of road engineering and water conservancy engineering, and these slopes will cause serious soil erosion under rainfall conditions. Because rainfall runoff is simultaneously affected by slope, bed surface and rainfall, the runoff movement characteristics are complex. It is difficult to consider all influencing factors in the existing models, especially for steep slopes. In this study, artificial rainfall experiments were conducted to study the rainfall-runoff hydraulic processes under different rainfall intensities and slope gradients, and a modified method was proposed to model the key hydraulic parameters (i.e., equilibrium time, water surface line, and runoff processes) on steep slopes. The results showed that (1) For steep slopes (a 70° slope compared to a 5° slope), the runoff generation time, confluence time and equilibrium time of the slope decreased significantly. At the same time, the single width runoff of the steep slope had a power function relationship with the rainfall intensity and gradient. (2) The runoff patterns of steep slopes were different from those on gentle slopes and runoff patterns were more likely to change. The Reynolds number and Froude number for slope flow changed slowly when the slope was less than the critical gradient and increased significantly when the slope exceeded the critical gradient. (3) Based on the analysis of the “double turbulent model theory of thin-layer flow on a high-steep slope”, combined with the dispersed motion wave model, a modified method for calculating the hydrodynamic factors of rainfall runoff was proposed. Then, this method was verified with indoor and outdoor experiments. The research results not only have theoretical significance, but also provide a more accurate calculation method for the design of high and steep slopes involved in land treatment engineering, road engineering and water conservancy engineering.

## Introduction

The combination of arid climate, uneven precipitation, and the intense agricultural activities caused by low available cultivated land has caused soil erosion in the loess hilly gully region and resulted the high sediment load in the Yellow River^1–3^. To increase the available land area, large-scale land consolidation projects have been carried out in recent years, including the construction of 658 silt dams, 2364 hydraulic structures, 880 km of roads, and an investment of 270 million in 2014^4^.

A large number of high and steep loess slopes were formed in the project, and their safety has attracted wide attention^5,6^. These loess high and steep slopes are characterized as being long and steep, and some are greater than 55°. Because these slopes are steep and long, serious soil erosion can occur under rainfall conditions. Therefore, it is necessary to study and analyze the movement characteristics and process of rainfall runoff on high and steep slopes, and explore the movement law of rainfall runoff on high and steep slopes.

In terms of hydraulics, the overland flow on high and steep slopes is thin-layer flow with an unstable direction, with a depth of a few millimeters, or even less than 1 mm. Due to the influences of slope, rainfall intensity, surface condition and other factors, a study on thin-layer flows on slope surfaces is more difficult than studies on open- channel flows^7^.

In terms of runoff generation processes and runoff velocity distribution, the effect of discharge on the mean flow velocity was determined by flume tests on slopes ranging from 3°~10°, and the results showed the vertical velocity profile with the changes in bed morphology^8^. Flow velocities were correlated with the discharge and hydraulic radius but not with the slope^9^. Based on the flume tests using different rainfall intensities on slopes of 1.5° to 15°, the slope velocity was positively correlated with the slope gradient^10^. The process of runoff generation on a loess slope was studied by an artificial rainfall simulation experiment, and the comprehensive effects of rainfall intensity, slope gradient and slope length on the thin-layer runoff depth on a loess slope could be expressed as a three-dimensional linear empirical equation^11,12^. According to the results of the runoff in the deposit body from 24° to 32°, the flow velocity was positively correlated with slope and discharge, and the influence of slope on flow velocity was greater than the influence on discharge^13^. Scholars at home and abroad have systematically studied the runoff generation process and velocity distribution on gentle slopes (<55°), while, the runoff generation processes on steep slopes have been less studied, and there is a lack of comparative analysis with that on gentle slopes.

In terms of hydraulic parameters and the flow pattern of slope runoff, overland flow may be wholly turbulent, wholly laminar, or partly turbulent and partly laminar-patches of laminar flow are interspersed with turbulent flow or vice versa patterns^14^. Although the slope flow disturbed by artificial rainfall had turbulent characteristics in experiments, it still exhibits multilayer flow characteristics^15^. The shallow open-channel flow on the slope is in the stable zone of layer loss during rainfall, and the flow pattern changes with variations in rainfall intensity and slope^7^. Drainage scouring and rainfall experiments were carried out to explore the effects of rainfall intensity and roughness on the hydrodynamic characteristics of slope flow, and the results showed that the thin layer of water flow was in the laminar flow and the flow area of the transition flow, additionally, as the slope increased, the shallow flow had a shifting trend from laminar flow to transition flow^16^. The divergence in the study of the Reynolds number was the definition of its laminar flow, and rainfall disturbance was the main reason for the uniqueness of its flow pattern^17,18^. To determine the effects of the spatial distribution of vegetation on rainfall runoff resistance and erosion processes, experiments were carried out from 3°~12°, and the results showed that grass roots could reduce soil erodibility and increase critical shear stress^19^. An indoor experiment was carried out to study the hydraulic characteristics of loess slopes (25°~50°) and their relationships, including the velocity, Reynolds number, Froude number and so on. The results showed that the rainfall intensity, gradient and their interactions had significant effects on the sheet flow hydraulic parameters^20^. The slope flow formed by rainfall was both transitional and turbulent, and was unstable and non-uniform in terms of its spatial and temporal distributions; thus, it was a disturbed flow that was seriously affected by the underlying surface and rainfall and had the characteristics of a jet flow^21,22^. Studies on the characteristics of runoff parameters on gentle slopes are more systematic, while studies on runoff of steep slope are limited, which leads to a lack of unified conclusions on the flow patterns of steep slopes, and a lack of the change rules for hydraulic parameters of rainfall runoff with different slopes.

**Figure 1:**
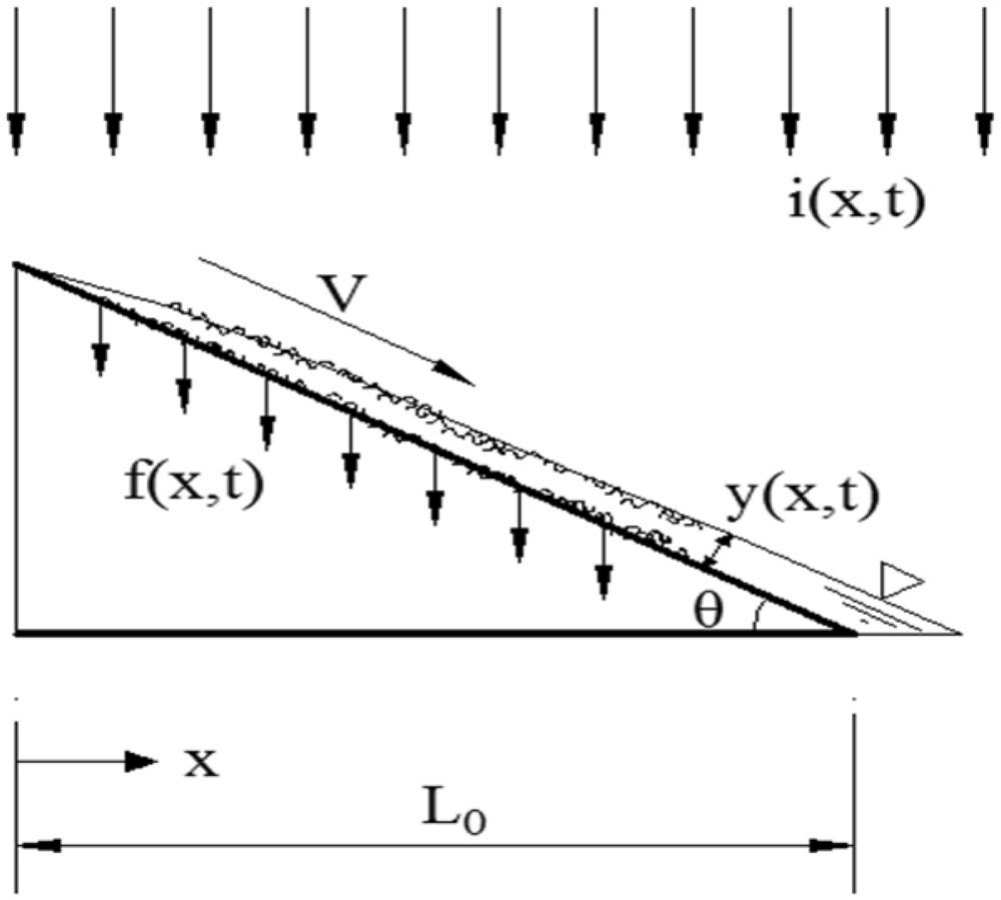
Schematic slope flow diagram on a high and steep slope.

In the slope runoff calculation process, the motion process of a one-dimensional unsteady plane flow is analyzed. Based on the power wave model^23^, three non-dimensional flow equations describing the increasing slope flow process are proposed. Dimensionless ascent curves are used for land surface flow, and highly accurate results can be obtained when the slope is small; additionally, the hydrodynamic parameters of slope flow from 1° to 5° can be calculated by using the method given by Ligget, J.A. and Woolhiser, D.A^24^. The calculated results are in accordance with the actual situation and can meet the actual production requirements^25^. Whether this method is suitable for steep slopes and how accurate it is requires more research. Gao first proposed a double-turbulent sheet flow model for high and steep slopes in the study of “a simulation study on the variation characteristics of a rainfall-runoff erosion flow on a complex underlying surface”. The schematic diagram of the model is shown in Fig. 1.

In Fig. 1, V is the flow velocity of the slope, m·s^−1^; i (x, t) is the rainfall intensity, and the function of x and t, m·s^−1^; f (x, t) is the infiltration intensity, and the function of x and t, m·s^−1^; y(x, t) is the flow depth of the slope, and the function of x and t, m; θ is the slope gradient; L_0_ is the length of the slope, m.

Figure 1 shows that the runoff on the high and steep slope was affected by the double turbulence, which were raindrop strikes and a rough bed surface. Raindrops formed by rainfall struck the upper surface of the runoff, and rough wounds affected runoff movement at the bottom of the runoff. The turbulence caused by the raindrop strike and flow movement diffused to the bed surface, while the turbulence on the bed surface diffused to the water surface. This bidirectional diffusion led to runoff movement on the high and steep slopes, which was different from the open-channel flow. On a high and steep slope, the effect of this influence on runoff should be considered.

In this paper, a large amount of domestic and international experimental data were systematically collated and analyzed. Through indoor and outdoor simulated rainfall experiments, the hydraulic characteristics, runoff generation and confluence processes of steep slopes with different rainfall intensities were studied. Based on the “double turbulent sheet flow theory of a high-steep slope” and the existing dispersed wave model, combined with rainfall experiments under mild slope conditions, the hydrodynamic parameter calculation processes in different rainfall intensities and different slope degrees were given. The results were preliminarily verified, which provided a scientific basis for safe erosion reduction and the ecological protection of high and steep loess slopes.

## Materials and methods

### Test materials

**Figure 2:**
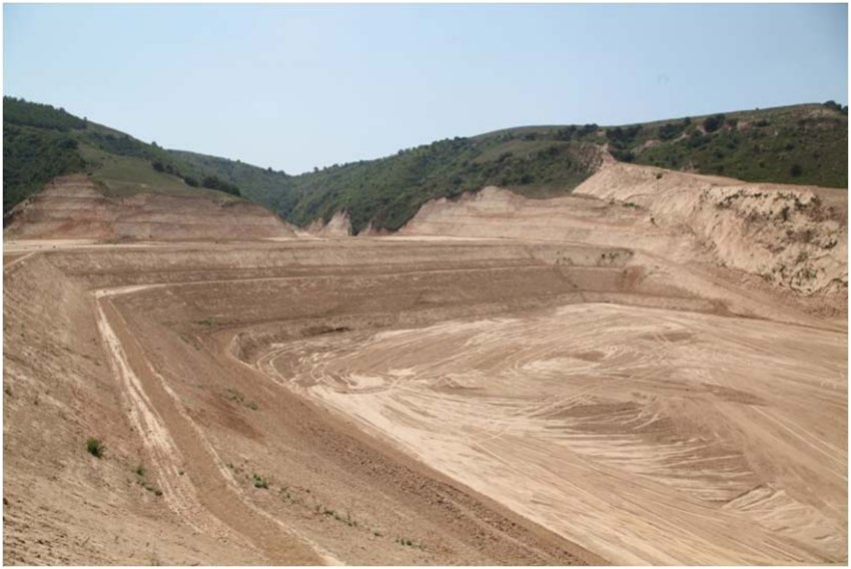
The No. 1 dam in Louping Township.

**Figure 3:**
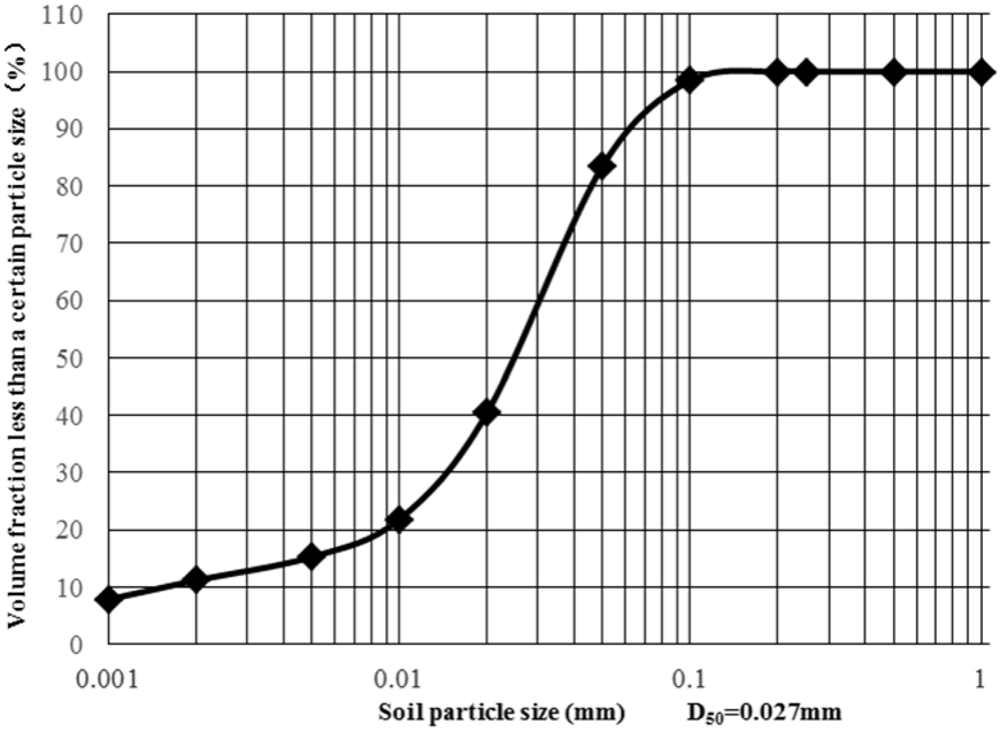
The mechanical composition of experimental loess.

The test loess was selected from the No. 1 dam construction site in Louping Township, Ansai County, Yan’an city, Shaanxi Province. The No. 1 dam is shown in Fig. 2, and the soil mechanical composition is shown in Fig. 3.

**Figure 4:**
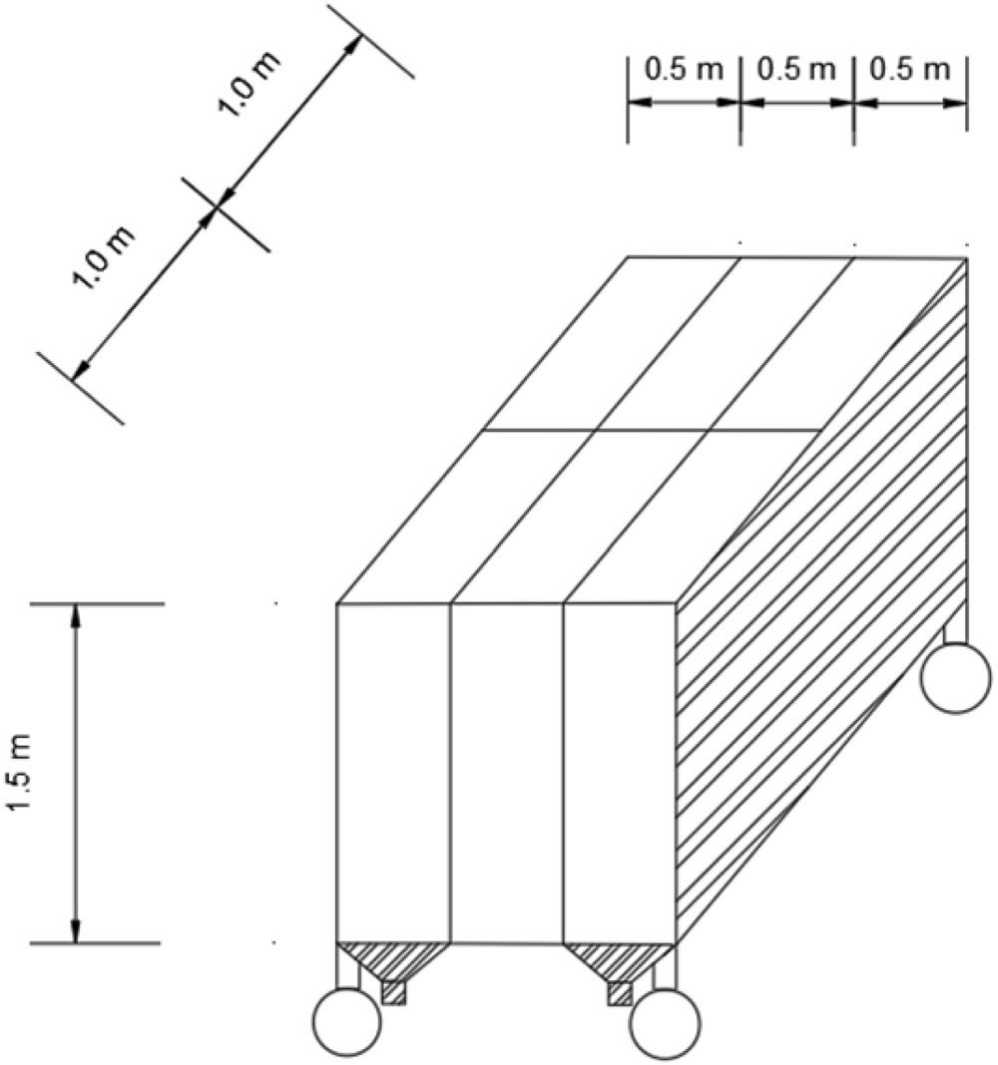
Soil tank for the rainfall-drainage simulated test.

**Figure 5:**
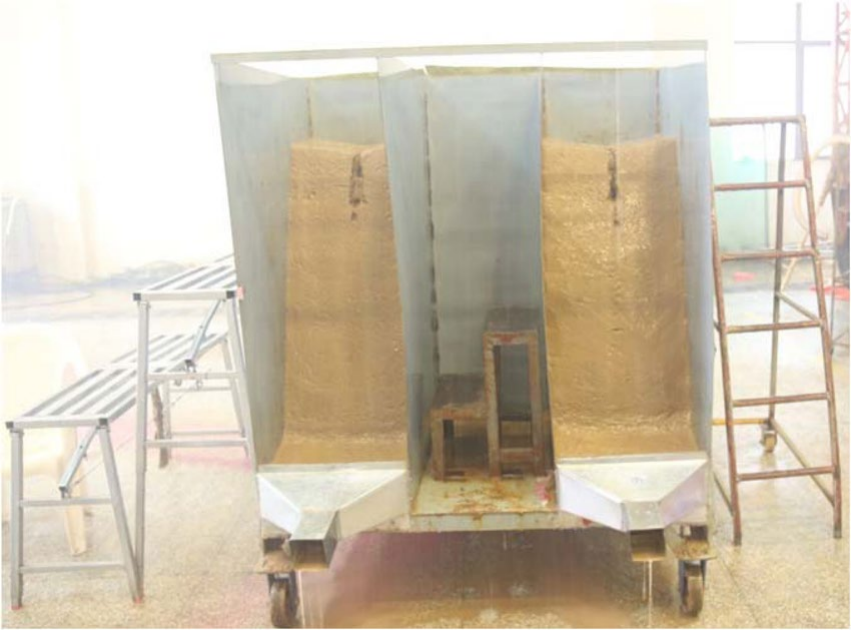
Simulated rainfall process.

The movable steel trough was independently designed by the research group. The size of the soil trough was 2.0 m × 1.5 m × 1.5 m, and was divided into six independent units, each of which was 1.0 m × 0.5 m × 1.5 m. Each lattice could carry out an independent rainfall test. Details of the test soil tank are shown in Fig. 4, and simulated rainfall process is shown in Fig. 5.

### Artificial rainfall simulations

The experiment was carried out in the artificial rainfall simulation hall at the Institute of Soil and Water Conservation, Ministry of Water Resources, Chinese Academy of Sciences^26^. Based on field sampling results of different positions in the No. 1 dam construction site, two kinds of high and steep slopes with different soil bulk densities (1.4 g·cm^−3^ and 1.5 g·cm^−3^) were designed. Through querying and calculating rainfall intensity in different periods based on rainfall data and the hydrological manual in Yan’an city, and considering the simulated situation in the rainfall hall, artificial rainfall intensities were selected at different gradients, which values of 30–135 mm·h^−1^ with 15 mm·h^−1^ step. The discharge volume was confirmed by the single-width discharge generated by rainfall in the experimental plot. The total amount of each rainfall event was controlled at 120 mm.

A lateral spray rainfall system was used in the experiment. The rainfall height was 18 m and the uniformity was over 80%. Before the test, the test soil tank was covered with a rain-shield cloth and the rainfall intensity was calibrated. One rain gauge cylinder was placed around the test tank to measure the rainfall. The standard error of rainfall intensity measured by four rain gauge cylinders was controlled at less than 10%, and the error of the calibration results and the designed rainfall intensity was less than 5%.

### Methods and statistical analysis

#### Methods

When the rainfall intensity is calibrated, the canopy should be uncovered, and when the slope begins to produce flow, the time should be recorded; the measurement, timing and sampling should begin subsequently.

First, the temperature of the water flow was measured by a thermometer to calculate the flow viscosity coefficient. The flow velocity and depth were measured regularly after the start of rainfall. During the test, the runoff and sediment process samples were collected every 3~5 min. After the test, the volume of the muddy water sample was measured, and the water sample was maintained for 8 h. The clear water on the upper part of the water sample was poured out, and the remaining sand sample was put into the drying box for drying and was then weighed.

### Statistical analysis

#### Velocity of flow

The velocity of flow was measured by the dye potassium permanganate tracer method, and the average value of three measurements was taken.

#### Average water depth

It is difficult to measure the depth of runoff under high and steep slope conditions. In this paper, the average water depth was calculated by the average velocity and discharge of each section in each period.

1 $${\rm{h}}=\frac{q}{V}$$

Where h is the depth of runoff, m. q is discharge of each section in each period, m^3^·s^−1^. V is the average velocity, m·s^−1^.

#### Reynolds number and froude number

The Reynolds number (Re) and Froude number (Fr), both of which are dimensionless, were the important bases for judging the flow pattern of the slope. The expressions were as follows:

2 $${\rm{Re}}=\frac{Vh}{\nu }$$

3 $${\rm{Fr}}=\frac{V}{\sqrt{gh}}$$

*ν* is the hydrodynamic viscosity coefficient, m^2^·s^−1^, $\nu =\frac{0.01775}{1\,+\,0.0337{\rm{t}}\,+\,0.000221{t}^{2}}$, t is water temperature, °C.

## Results and Discussion

Rainfall and roughness simultaneously affect the rainfall runoff of a high steep slope, which results in a change in the movement process of the rainfall runoff on high steep slopes, thus affecting the analysis and calculation of the movement process^25^. Therefore, based on the “theory of high steep slope double turbulent thin layer flow” and the existing dispersion wave model, the calculation process of runoff dynamic parameters with different rainfall intensities and slopes is given.

### Variations caused by double turbulence sources

**Figure 6:**
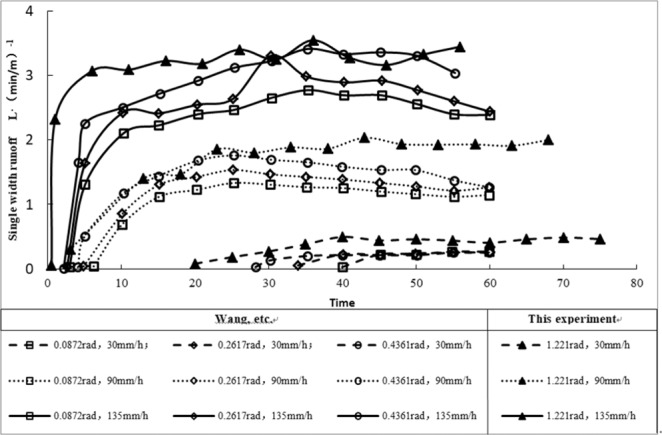
Runoff generation on slopes with different gradients.

The hydraulic characteristics of rainfall runoff on high and steep slopes were obviously different from those on gentle slopes. Figure 6 illustrates the runoff generation processes on slopes with different gradients published by this experiment and different scholars^21^. Figure 6 shows that the net discharge per unit width was positively correlated with rainfall duration during the process of runoff generation and in the confluence of slopes with different gradients. Moreover, the time of slope runoff generation varied with slope gradient. The larger the slope was, the smaller the starting time and confluence time were, and the larger the corresponding peak flow was.

For the same slope, the larger the rainfall intensity was, the smaller the starting time and confluence time were, and the larger the corresponding peak discharge was. For example, under the condition of 1.221 rad, the starting time of the 135 mm·h^−1^ rainfall intensity was approximately 7% of the 30 mm·h^−1^ rainfall intensity time, and the net flow per unit width of the 135 mm·h^−1^ rainfall intensity was approximately 520% of the 30 mm·h^−1^ rainfall intensity time.

Under the same rain intensity conditions, the greater the slope was, the shorter the starting time was, the shorter the equilibrium time was, and the larger the net flow per unit width of the slope surface was. For example, under the condition of 135 mm·h^−1^, the starting time of 1.22 rad was approximately 16% of the 0.087 rad condition. The net discharge per unit width of the 1.22 rad slope was approximately 144% of the 0.087 rad slope.

The runoff generation process of high and steep slopes was similar to that of gentle slopes, but it was more severely affected by the slope and rainfall intensity.

#### Variations in the Reynolds number with different rainfall intensities and slopes

**Figure 7:**
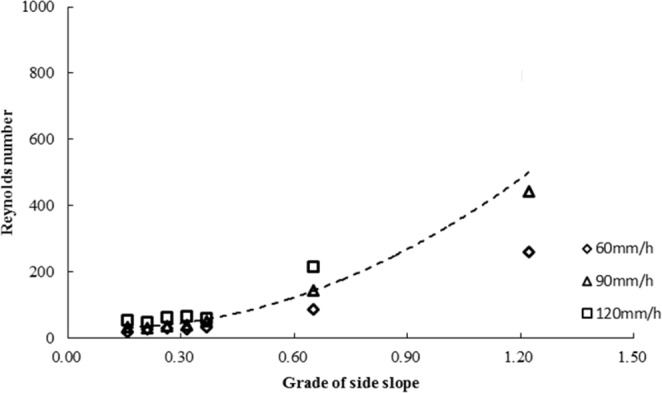
Variation in the Reynolds number with different rainfall intensities and slopes.

The Reynolds number is a parameter used to distinguish the flow pattern. Figure 7 shows that the Reynolds number on a high-steep slope was significantly positively correlated with rainfall intensity (60~120 mm·h^−1^). Compared with gentle slopes, the Reynolds number increased rapidly after runoff generation on steep slopes. With an increase in simulated rainfall intensity in the same soil bulk density and slope gradient conditions, the Reynolds number increased gradually, which indicated that an increase in rainfall intensity would change the slope flow pattern and would increase the degree of flow turbulence.

With the same rainfall intensity, when the slope was smaller than the critical slope, the Reynolds number did not change much; however, when the slope exceeded the critical slope, the Reynolds number increased rapidly. The empirical relationship between the Reynolds number and slope was as follows:

$${\rm{Re}}=376{{\rm{\theta }}}^{2}-80{\rm{\theta }}+35$$

With different rainfall intensities, the critical slope gradient with the obvious variation in the Reynolds number was approximately 0.42 rad.

#### Variation in the Froude number with different rainfall intensities and slopes

**Figure 8:**
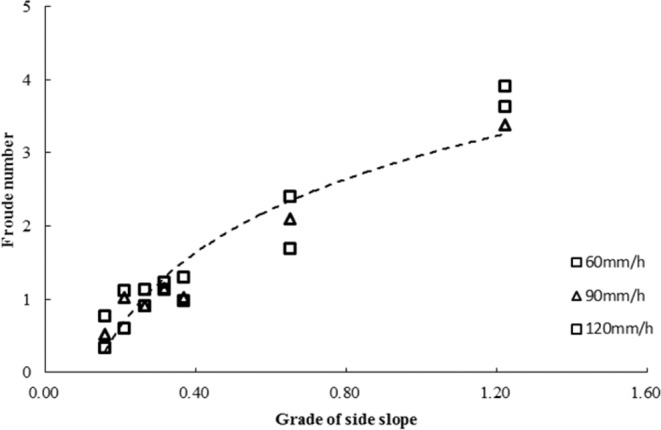
Variation in the Froude number with different rainfall intensities and slopes.

The Froude number, which is the ratio of inertial force and gravity in fluid, is a dimensionless number that is used to judge the state of the water flow. Figure 8 shows the Froude numbers with different rainfall intensities and slopes. With an increase in simulated rainfall intensity with the same soil bulk density, the Froude number increased gradually, and the Froude number after stabilization was greater than 1, which showed that the flow pattern would change with the increase in simulated rainfall intensity.

After runoff generation on steep slopes, the Froude number increased rapidly when the gradient did not exceed the critical gradient, and the runoff on slopes was rapid. When the critical gradient was exceeded, the Froude number tended to decrease with increasing slope. The empirical relationship between the Froude number and slope was as follows:

$${\rm{Fr}}=1.45\,\mathrm{ln}\,{\rm{\theta }}+297$$

With different rainfall intensities, the critical slope gradient at which the growth rate of Froude number obviously slowed was approximately 0.93 rad.

### Prediction of the hydraulic parameters

**Figure 9:**
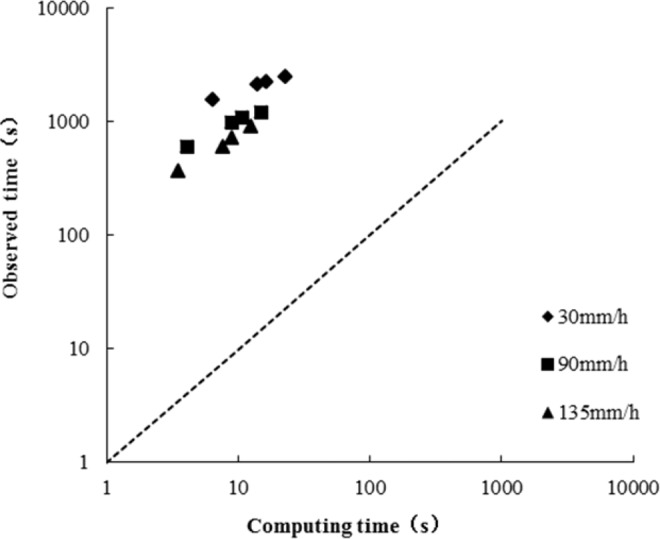
Comparisons of equilibrium times and computing times of steep slopes.

**Figure 10:**
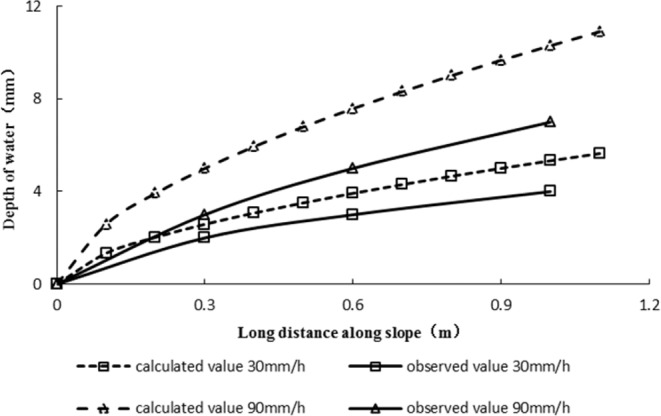
Comparisons of calculated and observed surface lines with different rainfall conditions.

**Figure 11:**
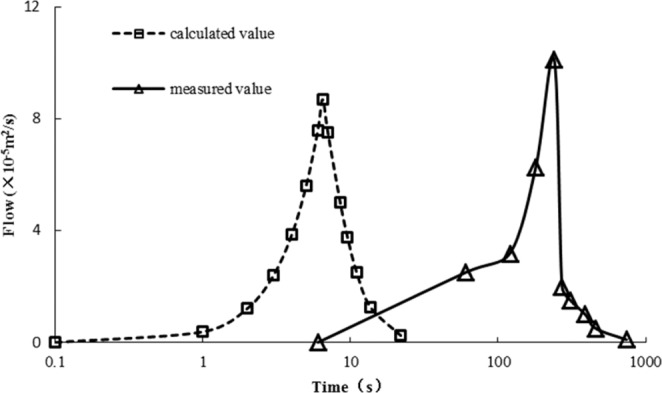
Comparisons between the calculated and observed outflow processes.

In practice, the calculation of hydrodynamic parameters of high and steep slopes directly affected the design of erosion and corrosion prevention engineering. Using the method given by Ligget, J.A. and Woolhiser, D.A^24^, Gao *et al*. calculated the hydrodynamic parameters of slope flow of 0.0174 rad and 0.0872 rad^25^. The calculated results were in accordance with the *in situ* data and met the actual production requirements. To determine the application of this method on high and steep slopes, the rainfall runoff on high and steep slopes was calculated. The calculation results are shown in Figs. 9–11.

#### The equilibrium time

Figure 9 shows that the calculation results of the slope flow equilibrium times of 0.26–1.22 rad were smaller, and the difference between the observed and calculated values was approximately 50-fold.

#### Water surface line

After calculating the runoff of high and steep slopes by the dimensionless method, the runoff water surface lines on high and steep slopes with different rainfall intensities could be calculated. Figure 10 shows that under the condition of high and steep slopes, the calculated value of the water surface line with different rainfall conditions was larger than the observed value, and the error along the slope length increased gradually; furthermore, the error at the foot of the slope was the largest, which was approximately 30% smaller than the calculated value.

#### Runoff and drainage on the slope surface

After calculating the runoff of high and steep slopes by the dimensionless method, the runoff outflow process of high and steep slopes with different rainfall intensities could be calculated. According to the runoff calculation process and actual outflow process of slopes, Fig. 11 was obtained. Figure 11 shows that there was a great difference in time between the calculated flow process and the observed flow process.

These verification results showed that when the original calculation method for the slope flow hydrodynamic parameters was used to calculate the high and steep slope, the calculated values of the slope flow equilibrium time, the slope flow water surface line and the slope outflow process were quite different from the observed values. Therefore, the equations must be modified when applying this method to the calculations of high and steep slopes.

### Model building

Compared with open-channel flows, the slope flows had the following characteristics: there was no fixed boundary for the slope flow; the slope flow depth was far less than the open-channel flow; and the influences of rainfall, infiltration and roughness on the slope flow were more obvious than those in the open-channel flow. In most cases, the slope flow movement was approximately described by the motion wave model^23^. Because of the large slope and the previously described influence of rainfall, the high and steep slope had runoff and was considered “sheet flow influenced by double turbulence sources”. Therefore, the surface flow movement on the high and steep slopes had a significant variation compared with the gentle slope. In the calculation of the high and steep slope surface flow, we had to consider the effects of slope gradient and rainfall at the same time.

On the slope, if the flow direction was the x-axis and the slope length was L, the Saint-Venant equation group of a one-dimensional unstable flow controlling the movement of slope flow was as follows:

4 $$\frac{\partial ({{\boldsymbol{V}}}_{{\boldsymbol{y}}})}{\partial {\boldsymbol{x}}}+\frac{\partial {\boldsymbol{y}}}{\partial {\boldsymbol{t}}}=i(x,t)-f(x,t)=r(x,t)$$

5 $${{\boldsymbol{S}}}_{0}-{{\boldsymbol{S}}}_{{\boldsymbol{F}}}=\frac{\partial {\boldsymbol{y}}}{\partial {\boldsymbol{x}}}+\frac{1}{{\boldsymbol{g}}}\frac{\partial {\boldsymbol{V}}}{\partial {\boldsymbol{t}}}+\frac{{\boldsymbol{V}}}{{\boldsymbol{g}}}\frac{\partial {\boldsymbol{V}}}{\partial {\boldsymbol{x}}}+\frac{{\boldsymbol{V}}}{{\boldsymbol{gy}}}[{\boldsymbol{i}}({\boldsymbol{x}},{\boldsymbol{t}})-{\boldsymbol{f}}({\boldsymbol{x}},{\boldsymbol{t}})]$$

When the international standard units were used in the equations, y was the flow depth of the slope, m; V was the flow velocity of the slope, m·s^−1^; i(x, t) was the rainfall intensity, and the function of x and t, m·s^−1^; f (x, t) was the infiltration intensity, and the function of x and t, m·s^−1^; r(x, t) was the net rainfall intensity, and the function of x and t, m·s^−1^; r(x, t) = i(x, t) − f(x, t), m·s^−1^; S_0_ was the slope gradient; S_F_ was the friction gradient; and g was the gravity acceleration.

In a classical study of the slope flow problem^27–29^, the slope gradient was small (less than 5°) when considering that slope gradient i_0_ and resistance slope gradient i_f_ were approximately equal:

6 $$\,{i}_{0}-{i}_{f}=0$$

In this case, Eqs. (4) and (5) were simplified, and the slope flow was regarded as a uniform flow. In the motion wave model, the slope was equal to the resistance slope. With the small slope, the motion wave model analyzed the slope flow more accurately^25^. When the actual slope exceeded 5°, Eq. (6) was no longer valid, as the original motion wave model calculation had errors. Therefore, the influences of slope and rainfall on the accuracy of the model should be considered in the analytical calculation of a large slope flow.

#### Correction of equilibrium time

An equation for calculating the equilibrium time was obtained based on the motion wave theory (7):

7 $${t}_{e}=\frac{1}{{r}^{0.4}}{\left(\frac{n{L}_{0}}{{{S}_{0}}^{0.5}}\right)}^{0.6}$$

Where t_e_ is the equilibrium time, s; r is the net rainfall intensity, m·s^−1^; n is roughness; L_0_ is the slope length, m; S_0_ is the slope, rad.

On a high and steep slope, M_1_ stands for the influences of the bed surface and rainfall on the variation in equilibrium time, and then Eq. (7) becomes:

8 $${t}_{em}={M}_{1}\frac{1}{{r}^{0.4}}{\left(\frac{n{L}_{0}}{{{S}_{0}}^{0.5}}\right)}^{0.6}$$

Based on the observed data, the relationship expression of M_1_ was obtained as follows:

9 $${M}_{1}=\frac{{S}_{0}}{{i}^{0.4}\theta }$$

Equation (9) shows that the influences of slope and rainfall on the equilibrium time are important. By substituting Eq. (8), a unified relationship of the equilibrium time with different gradients was obtained:

10 $${t}_{e}=\frac{{S}_{0}}{{i}^{0.4}\theta }\frac{1}{{r}^{0.4}}{\left(\frac{n{L}_{0}}{{{S}_{0}}^{0.5}}\right)}^{0.6}$$

Equation (10) was an equation for calculating the runoff equilibrium time on slopes, and the equation showed that the runoff equilibrium time on high and steep slopes was affected by the bed roughness, rainfall intensity and slope.

#### Correction of water surface line

Similarly, based on the motion wave theory, the equation for calculating the flow surface line on slopes was obtained (11):

11 $$y={\left(\frac{nxr}{{{S}_{0}}^{0.5}}\right)}^{0.6}$$

Where y is the water depth on the slope, x is the coordinate along the journey, and the other parameters have the same meanings as described above.

On a high and steep slope, M_2_ stands for the influence of the bed surface and rainfall on the variation in the flow surface line on the slope surface, and Eq. (11) becomes:

12 $$y={M}_{2}{\left(\frac{nxr}{{{S}_{0}}^{0.5}}\right)}^{0.6}$$

Based on the observed data, the relationship expression of M_2_ was obtained as follows:

13 $${M}_{2}=\frac{{\theta }^{0.6}}{{S}_{0}^{1.5}{i}^{0.1}}$$

Equation (13) shows that the influences of slope and rainfall on the water surface line are important. By substituting Eq. (11), the unified relationship of the flow surface lines on slopes with different gradients was obtained:

14 $$y=\frac{{\theta }^{0.6}}{{S}_{0}^{1.5}{i}^{0.1}}{\left(\frac{nxr}{{{S}_{0}}^{0.5}}\right)}^{0.6}$$

Equation (14) is an equation for calculating the runoff surface line on slopes, and the equation shows that the distribution of the rainfall runoff water surface line on high and steep slopes is affected by bed roughness, rainfall intensity and slope.

#### Outflow process modification

Figure 11 shows that the calculated flow process of slopes under different rainfall conditions is very short on a high and steep slope. The observed flow process time was approximately 50 times higher than the calculated time. Being affected by the slope gradient and rainfall, the equilibrium time and water surface line of the steep slope had to be modified. The steep slope flow process was obtained from the corrected equilibrium time and equilibrium flow at the foot of the slope.

Based on our analysis, in considering the influences of the slope gradient and rainfall intensity on the runoff flow, the original calculation process was modified. The modified equations and the original equations are shown in Table 1, including the equilibrium time, the slope surface line, and the rising and retreating processes of the slope flow.

**Table 1:** Slope Flow Original Calculation Process and Modified Calculation Process.

Slope Flow Parameters	Original equations	Modified equations
Motion Parameter	${K}_{1}=g{n}^{1.2}{{S}_{0}}^{0.4}{{L}_{0}}^{0.2}{r}^{-0.8}$	${K}_{2}=g{n}^{1.2}{{S}_{0}}^{0.4}{{L}_{0}}^{0.2}{r}^{-0.8}$
Equilibrium time	${t}_{e1}=\frac{1}{{r}^{0.4}}{\left(\frac{n{L}_{0}}{{{S}_{0}}^{0.5}}\right)}^{0.6}$	${t}_{e2}=\frac{{S}_{0}}{{i}^{0.4}\theta }\frac{1}{{r}^{0.4}}{\left(\frac{n{L}_{0}}{{{S}_{0}}^{0.5}}\right)}^{0.6}$
Water surface line	${y}_{1}={\left(\frac{nxr}{{{S}_{0}}^{0.5}}\right)}^{0.6}$	${y}_{2}=\frac{{\theta }^{0.6}}{{S}_{0}^{1.5}{i}^{0.1}}{\left(\frac{nxr}{{{S}_{0}}^{0.5}}\right)}^{0.6}$
Balanced flow at foot of slope	${q}_{e1}={L}_{0}r$	${q}_{e2}={L}_{0}r$
Rising process at foot of slope	${q}_{1}=r{L}_{0}{\left(\frac{t}{{t}_{e1}}\right)}^{5/3}$	${q}_{2}=r{L}_{0}{\left(\frac{t}{{t}_{e2}}\right)}^{5/3}$
Receding process at foot of slope	${t}_{1}=\frac{{t}_{e1}}{m}{\left(\frac{q}{{q}_{e1}}\right)}^{1/m}\left(\frac{{q}_{e}}{q}-1\right)$	${t}_{2}=\frac{{t}_{e2}}{m}{\left(\frac{q}{{q}_{e2}}\right)}^{1/m}\left(\frac{{q}_{e}}{q}-1\right)$

In Table 1, K is the motion parameter; t_e_ is the slope flow equilibrium time, s; y is the depth of the flow, m; q_e_ is the single wide flow when slope flow reached equilibrium, m^2^·s^−1^; t is rainfall duration, s; n is roughness; L_0_ is the length of slope, m; r is the net rainfall intensity, m·s^−1^; m is empirical constant; i is the rainfall intensity, m·s^−1^; θ is the slope gradient.

Table 1 shows the original calculation process and modified calculation process of flow on slopes. In Table 1, the motion parameter(K_1_ and K_2_) is same and is used as the discriminant number of flow. Balanced flow at foot of slope(q_e1_ and q_e2_) is just affected by rainfall intensity under the certain slope length. Equilibrium time, water surface line, rising and receding process at foot of slope are all affected by the double-turbulent, and the influences of the bed surface and rainfall on the variation are shown in the new equations.

### Performances of the modified methods

#### Equilibrium time verification

**Figure 12:**
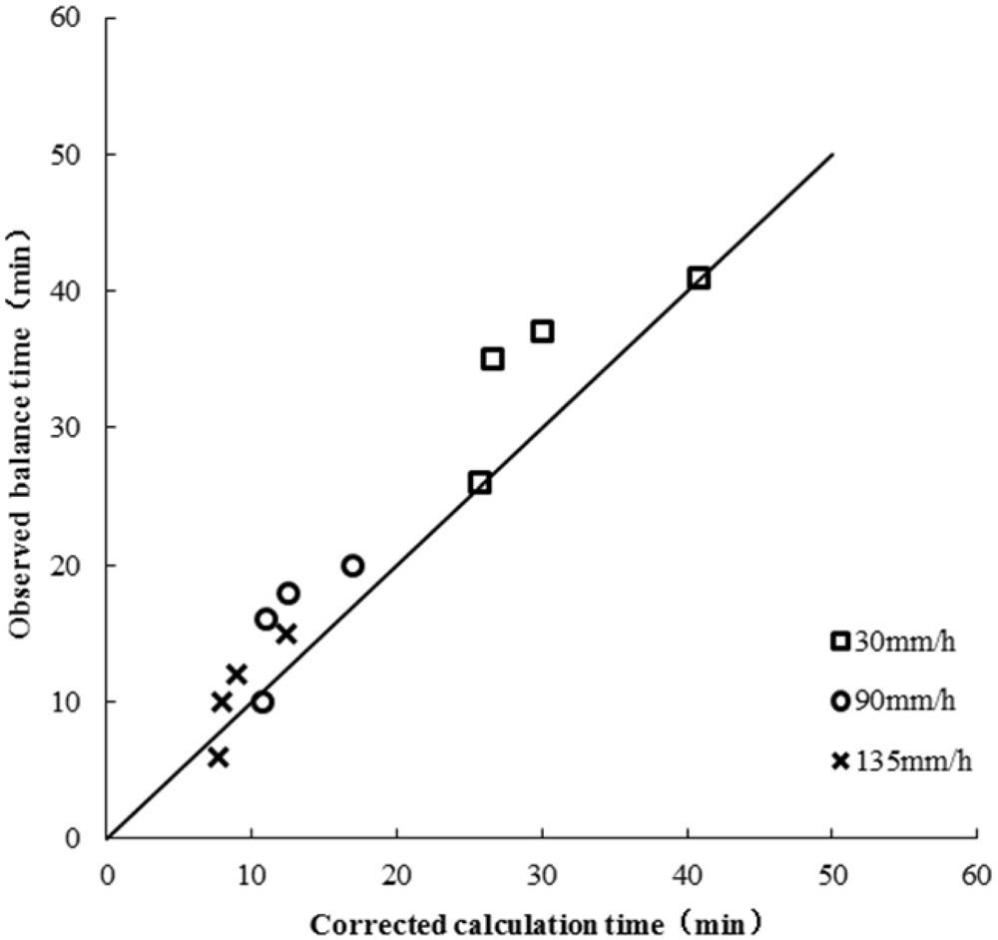
Calculated and observed values of equilibrium time.

Based on the observed data of indoor and field slopes with different rainfall intensities of 0.087 rad, 0.26 rad, 0.44 rad, 0.65 rad and 1.22 rad, the runoff equilibrium time on slopes was calculated by using the modified equations. The calculated runoff equilibrium time on slopes was compared with the observed values as shown in Fig. 12.

Figure 12 shows that using the modified methods, the difference between the calculation equilibrium time and the measured equilibrium time under different conditions is small, and the effect is good.

#### Verification of the water surface line

**Figure 13:**
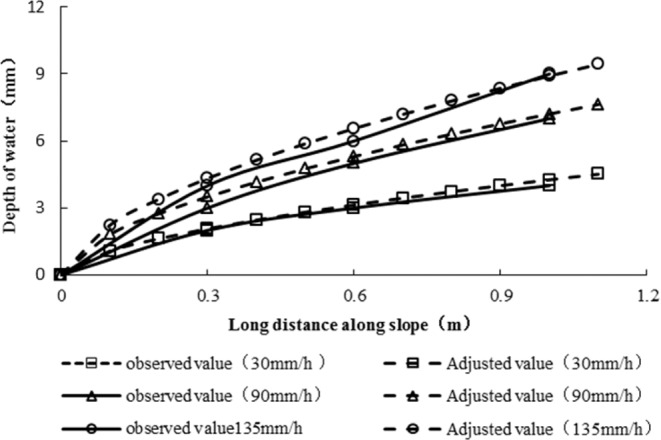
Relationship diagram between the observed water surface lines and calculated water surface lines.

According to the observed water surface line in the test and the adjusted calculated water surface line, the water surface line of the high and steep slope was obtained as shown in Fig. 13. As shown in Fig. 13, the adjusted calculated water surface line along the slope length was essentially the same as the observed water surface line.

Figure 13 shows that using the modified methods, the shape of the calculated water surface line is similar to the observed ones, and the depth of the calculated value and the measured value does not exceed 10%.

#### Outflow process verification

**Figure 14:**
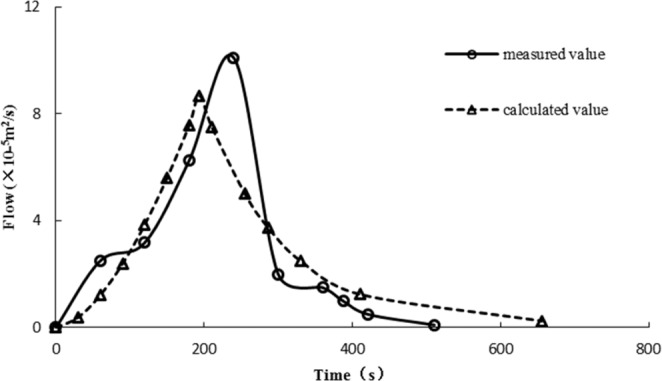
Calculated balance time after adjustment and observed values of the laboratory test.

**Figure 15:**
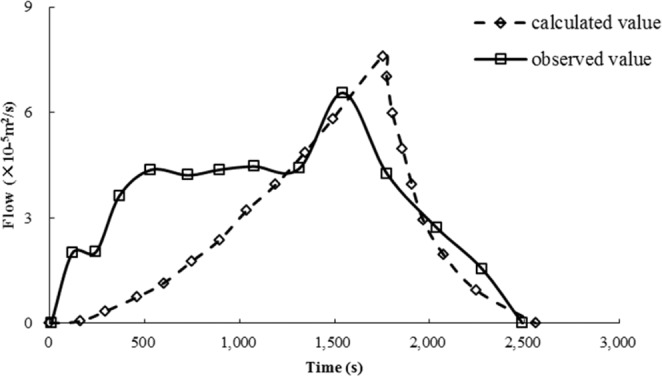
Calculated balance time after adjustment and field measurements.

According to the observed outflow process indoor test, field observations and the adjusted outflow process, the high and steep slope outflow process was obtained as shown in Figs. 14 and 15. From Figs. 14 and 15, the calculated flow process of the adjusted steep slope was similar to the results of the laboratory tests and field observations.

Figures 14 and 15 show that using the modified methods, the duration of the calculated rising process and the calculated receding process are similar to the observed ones, and the flow rates at different times are not much different, and the balance flow at the foot of the slope is basically the same.

These verification results showed that the calculated values of the parameters such as the equilibrium time, water surface line and the slope flow outflow process were in accordance with the observed values. The modified equations were able to apply in the calculation of the high and steep slope flow.

## Conclusion

Through our analysis, the following conclusions were drawn:

1. Compared with gentle slopes, the runoff yield-confluence time and equilibrium time of the steep slope decreased, and the single width runoff of the steep slope had a power function relationship with the rainfall intensity and slope, which was a 3/2 power relationship with the rainfall intensity and a 1/6 power relationship with the slope.
2. The slope flow pattern was influenced by both the slope and the rainfall intensity, and a critical gradient existed in the influence of slope on the flow pattern. The steep slope runoff varied from the gentle slope when the slope was over the critical gradient, the Reynolds number for the steep slope runoff was significantly higher than the gentle slope, and the runoff was more likely to become a supercritical flow on steep slopes.
3. The dispersed motion wave model proposed by the original hydraulic approach did not consider the sheet flow simultaneously affected by double turbulent sources, which led to poor calculation accuracy. In this study, based on the “double turbulent source sheet flow model of high and steep slopes”, a new calculation method was proposed and verified when combined with indoor and outdoor test data. The new model could be used to calculate hydraulic parameters of the slope runoff, and its accuracy was greatly improved, and it could be used for calculations of slope flow processes on high and steep slopes.
